# Histamine-binding capacities of different natural zeolites: a comparative study

**DOI:** 10.1007/s10653-018-0129-5

**Published:** 2018-06-07

**Authors:** Thangaraj Selvam, Wilhelm Schwieger, Wilfried Dathe

**Affiliations:** 1 Institute of Chemical Reaction Engineering, Friedrich-Alexander-Universität Erlangen-Nürnberg, Egerlandstraße 3, 91058 Erlangen, Germany; 2 Heck Bio-Pharma GmbH, Gerberstraße 15, 73650 Winterbach, Germany

**Keywords:** Natural zeolite, Cuban and Mexican zeolites, Clinoptilolite, Mordenite, Morphology, Histamine uptake

## Abstract

Two different natural zeolites from Cuba and Mexico, which are already being used as contemporaneous drugs or dietary supplements in Germany and Mexico, respectively, are applied in a comparative study of their histamine-binding capacities as a function of their particle sizes. The zeolites are characterized using X-ray diffraction (XRD), scanning electron microscopy (SEM) and N_2_-sorption measurements (BET surface areas). The Cuban zeolite contains clinoptilolite and mordenite as major phases (78% zeolite), whereas the Mexican one contains only clinoptilolite (65% zeolite). Both zeolites are apparently free from fibrous materials according to SEM. Both zeolites adsorb significant amount of histamine under the experimental conditions. Nevertheless, the results showed that the histamine-binding capacity of the Cuban zeolite is higher than the Mexican one and the smaller the particle size of zeolite, the higher the histamine-binding capacity. This difference could be due to the variation in their mineralogical compositions resulting in varied BET surface areas. Thus, the high histamine-binding capacities of Cuban zeolites seem to be due at least partly to the presence of the large-pore zeolite mordenite, providing high total pore volumes, which will be discussed in detail. For the first time, we have shown that the mineralogical compositions of natural zeolites and their particle sizes play a key role in binding histamine, which is one of the most important regulators in human physiology.

## Introduction

Natural zeolites are microporous crystalline aluminosilicates that possess well-ordered open pores in the form of regular channels and cages of molecular dimensions (< 1.3 nm), which have found wide range of applications including ion exchange, environmental pollution control, soil remediation and medicine (Inglezakis and Zorpas 2012). Over the past decades, there has been increased interest in applying natural zeolites as active ingredients in human and veterinary supplements. They are used either in oral supplements for endogenous well-being or in topical application for skin discomfort (Rodríguez-Fuentes et al. 1997, 2006; Andronikashvili et al. 2009; Colella 2011; Milić et al. 2014; Cerri et al. 2016; Dathe 2016), due to their excellent binding capacities for toxins and other harmful substances.

Histamine (2-[4-imidazolyl] ethylamine) is a biogenic amine of histidine and is a potent mediator of numerous biological reactions including histamine intolerance (Maintz and Novak 2007). The main metabolic pathway runs over diamine oxidase (DAO) which catabolizes ingested histamine, which is commonly found in various consumer products like wine, beer, cheese, sardine fillets, etc. (Sarkadi 2004). Impaired histamine degradation by reduced or blocked DAO activity or too high histamine levels may cause several painful reactions like headache, pruritus, diarrhea, etc. Thus, persons with low DAO activity are at risk of histamine toxicity (Maintz and Novak 2007; Westly 2010). On the other hand, histamine adsorption takes place by zeolite exclusively within the gastrointestinal tract, because of the large particle size of zeolite that does not permit the passage of the zeolite into the bloodstream (Selvam et al. 2014). Therefore, knowledge about some specific parameters (particle sizes and mineralogical compositions), which are involved in effective histamine-binding capacities, is very important for selecting a suitable natural zeolite for medical applications as well as to explain the observed clinical results. It is well documented in the literature that histamine plays an important role as a regulator for human physiological processes, such as allergic inflammation (Akdis and Simons 2006) and immune response (O’Mahony et al. 2011). Therefore, the main goal of these studies was to estimate the histamine uptake by natural zeolites having different mineralogical compositions and particle sizes.

Important clinical trials have been conducted with respect to the reduction of episodes of heartburn by zeolite clinoptilolite as well as the decrease of mucosa erosion under consumption of non-steroidal anti-inflammatory drugs (Potgieter et al. 2014). Symptoms of veisalgia caused by alcohol overindulgence can be significantly reduced by zeolite application without any effect on blood- or breath-alcohol level (Gandy et al. 2015). It is important to note that all these symptoms within the stomach or of veisalgia are strongly connected with the endogenous histamine level, among others, which is implicated in the pathogenesis of gastritis, headache, nausea, etc. In addition, food-borne pathogens are able to produce harmful agents, which can be reduced by zeolite clinoptilolite in order to prevent any type of risk surrounding consumer health (Özogul et al. 2016). The effect of zeolite on histamine production and accumulation in food-borne pathogens seems to be dependent on the bacterial species and zeolite concentration (Gokdogan et al. 2012).

The general aim of our investigations is to correlate the zeolite properties to biophysical and biochemical parameters in order to know which physiological processes could be influenced by zeolite. In our recent studies (Selvam et al. 2014), we have reported the thorough characterization (purity, mineral composition, ion exchange and histamine-binding capacities) of natural Cuban zeolite, which is known as a weak antacid and anti-diarrheic drug extensively tested with respect to its toxicological and pharmacological compatibility (Rodríguez-Fuentes et al. 1997, 2006; Rivera et al. 2003). In the present investigation, we focused the attention on the effects of particle size and mineralogical composition on the histamine-binding capacity. For this comparative study, we have used two natural zeolites from Cuba and Mexico having different mineralogical compositions and particle sizes, which are available as contemporaneous drugs and dietary supplements in Germany (Detoxsan^®^ Pulver) and Mexico (Zeolita Natural), respectively, in order to facilitate a comparison of their most important parameters.

## Experimental

### Materials

The zeolites used in the present study were procured from Cuba, San Andrés (particle sizes: ~ 3 and ~ 40 µm) and Mexico, Mina San Francisco, San Felipe, Guanajuato (particle size: ~ 20–150 µm). The suppliers of natural zeolites used conventional mechanical grinding process in order to obtain the natural zeolite powders (Cuban and Mexican). Note that the zeolites were used as received from the respective suppliers without further tribomechanical micronization and chemical or thermal treatments. Nevertheless, Mexican zeolites, having different particle size fractions, were collected by sieving method (See section: Characterization). Synthetic zeolites, such as clinoptilolite and mordenite, were obtained according to the standard literature procedures (Selvam and Schwieger 2002; Güvenir et al. 2009) and used for the quantification of zeolite phases (clinoptilolite and mordenite) that are present in natural zeolites. Note that synthetic clinoptilolite sample was kindly provided by Prof. Ali Çulfaz, Department of Chemical Engineering, Middle East Technical University, Ankara, Turkey. Histamine (97%) was purchased from Sigma-Aldrich.

### Characterization

In particular, particle size distribution analysis of the Cuban zeolite (~ 3 µm) was done at Micromeritics Analytical Services Europe, Aachen, Germany, in a Saturn DigiSizer II 5205 V1.04 (0.3 g of zeolite was dispersed in 20 mL 0.2% of sodium (hexa) metaphosphate in deionized water) using dynamic light scattering measurement. The particle size distributions of the Mexican (~ 20–150 µm) and Cuban zeolites (~ 40 µm) were carried out by sieving method (Weiner and Fairhurst 1992) using a vibratory sieve shaker (ANALYSETTE 3 PRO, Fritsch). A stack of 5 sieves (with apertures of 20, 40, 63, 100 and 150 µm) and a bottom plate was used in accordance with DIN EN 1015-1: 2007-05 (2007).

The X-ray diffraction (XRD) patterns of the samples were recorded on a Philips X-ray diffractometer using Cu-Kα radiation. The XRD patterns were collected in the 2θ range between 2° and 50° (step size: 0.03°; time per step: 10 s; total scan time: 270 min). The identification and quantification of the zeolite phases were carried out according to the previously published procedures (Selvam et al. 2014). The morphology of both the Cuban and Mexican zeolites was studied using an environmental scanning electron microscope (FEI Quanta 200). The BET surface areas and total pore volumes of the samples were determined by an automated nitrogen adsorption analyzer (Quantachrome Instruments) at 77 K. Prior to the sorption measurements, all the samples were pre-treated under high vacuum at 250 °C for 12 h.

### Histamine uptake studies

Histamine-binding studies of Cuban and Mexican zeolites, having different particle sizes, were carried out at pH = 7 and the amount of histamine adsorbed by natural zeolites was estimated by thermogravimetric measurements (TG–DTA) as described previously (Selvam et al. 2014). In a typical experiment, 3 g of the zeolite sample was dispersed in double distilled water (100 mL) in a polypropylene bottle (250 mL). Then, the histamine (0.3 g) was added to the above solution. The polypropylene bottle containing the mixture was closed tightly and shaken using a water bath (GFL 1083; 90 cycles per min) at 36 °C. The histamine uptake was monitored by taking small aliquots (~ 5 mL) from the mixture at different times (15, 30, 60 and 120 min), filtered, dried at room temperature for 24 h and the solid sample (histamine-loaded zeolite) was analyzed by TG–DTA analysis (TA instruments SDT 2960). The sample was heated up at a rate of 10 °C/min from room temperature to 900 °C under air atmosphere. The difference in weight loss between the histamine-loaded zeolite and the pure zeolite samples in the temperature range of 350–600 °C was considered for the amount of histamine uptake by the respective samples.

## Results and discussion

### X-ray diffraction (XRD)

**Fig. 1:**
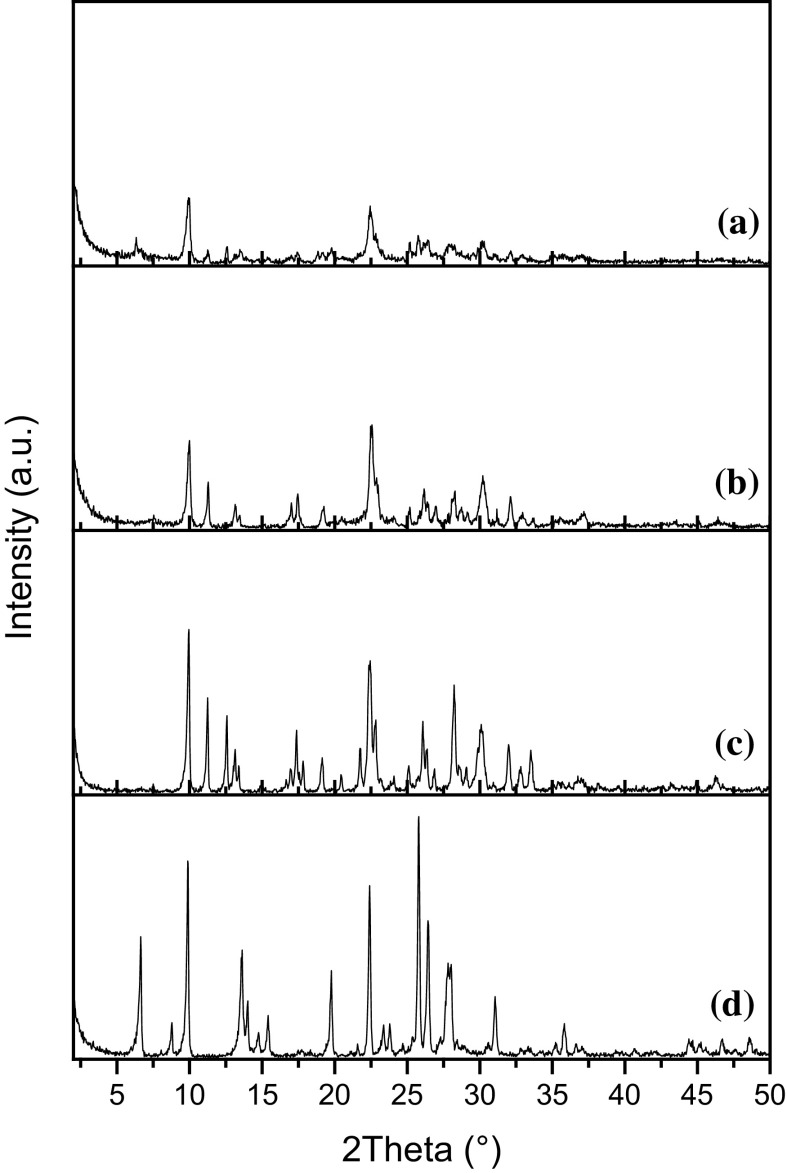
XRD patterns of the natural Cuban (~ 3 µm) (**a**) and Mexican (~ 20–150 µm) (**b**) zeolites. For comparison purpose, synthetic zeolites such as clinoptilolite (**c**) and mordenite (**d**) are also included

The XRD patterns of the natural Cuban (~ 3 μm) and Mexican (~ 20–150 µm) zeolites and synthetic zeolites, such as clinoptilolite and mordenite are displayed in Fig. 1. The XRD pattern of the Cuban zeolite (Fig. 1a) contains the characteristic diffraction peaks of both clinoptilolite and mordenite. All the peak positions and relative intensities match quite well with the synthetic zeolites clinoptilolite (Fig. 1c) and mordenite (Fig. 1d). According to the XRD results, no other impurity phases were detected. The XRD pattern of the Mexican zeolite (Fig. 1b) confirms the presence of clinoptilolite as the predominant phase. The absence of additional diffraction peaks, related to mordenite and/or other zeolite phases, indicates that both the Mexican and Cuban zeolites are pure and free from other impurities.

In addition, the XRD patterns of the synthetic zeolites allowed us to determine the semi-quantitative mineralogical compositions of both the natural Cuban and Mexican zeolites by calculating the ratio of the sum of the (integral) intensities of most intense peaks to those of phase-pure synthetic zeolites (clinoptilolite and mordenite). The mineralogical compositions of both the natural Cuban and Mexican zeolites are summarized in Table 1. The contents of zeolites present in both Cuban and Mexican zeolites are estimated to be around 78% (clinoptilolite: 43% and mordenite: 35%) for the Cuban and 65% (only clinoptilolite) for the Mexican zeolites, respectively. The estimated zeolite content of Cuban sample (78%; ~ 3 μm) is slightly less than the previous estimation (85%; ~ 40 μm) (Selvam et al. 2014). These small fluctuations could probably be due to the dust extraction system employed by the supplier to obtain fine-grained Cuban zeolite (~ 3 μm) during the conventional mechanical grinding process as well as to the inherent differences (in the zeolite grade) associated with natural deposits. Although nearly all the zeolite contents were identified in both the natural Cuban and Mexican zeolites, still there are remaining unidentified and/or amorphous phases (22–32%). This could be partly due to that fact that the XRD patterns of the natural zeolites (Fig. 1a, b) exhibit very broad and less intense peaks in comparison to the XRD patterns of the synthetic zeolites (Fig. 1c, d; very sharp and intense peaks) indicating the presence of X-ray amorphous materials, which are typically found in natural minerals. The latter fact could help to explain the small fluctuations in the zeolite content (78%; ~ 3 μm) in the fine-grained Cuban zeolite in comparison to previous estimations (85%; ~ 40 μm).

**Table 1:** Semi-quantitative estimation of the mineralogical compositions of the Cuban (~ 3 µm) and Mexican (~ 20–150 µm) zeolites

Zeolite	Mineralogical composition (%)^a, b, c^				
	Clinoptilolite	Mordenite	Quartz	Anorthite	Unidentified or amorphous phases
Cuban	43	35	–	–	22
Mexican	65	< 1	< 1	< 1	32

^a^Mineralogical compositions were calculated using synthetic zeolites (clinoptilolite and mordenite) as external references

^b^The phase contents of clinoptilolite and mordenite present in the Cuban and Mexican zeolites were estimated by calculating the ratio of the sum of the (integral) intensities of 8 most intense peaks to those of phase-pure synthetic counterparts

^c^The following set of {hkl}(2θ°) values was considered for clinoptilolite: {020}(9.88), {200}(11.19), {111}(17.36), {131}(22.36), {240}(22.82), {− 222}(26.04), {− 422}(28.15) and {151}(30.05); and mordenite: {110}(6.51), {200}(9.77), {111}(13.45), {330}(19.61), {150}(22.20), {202}(25.63), {350}(26.25) and {511}(27.67)

### Scanning electron microscopy (SEM)

**Fig. 2:**
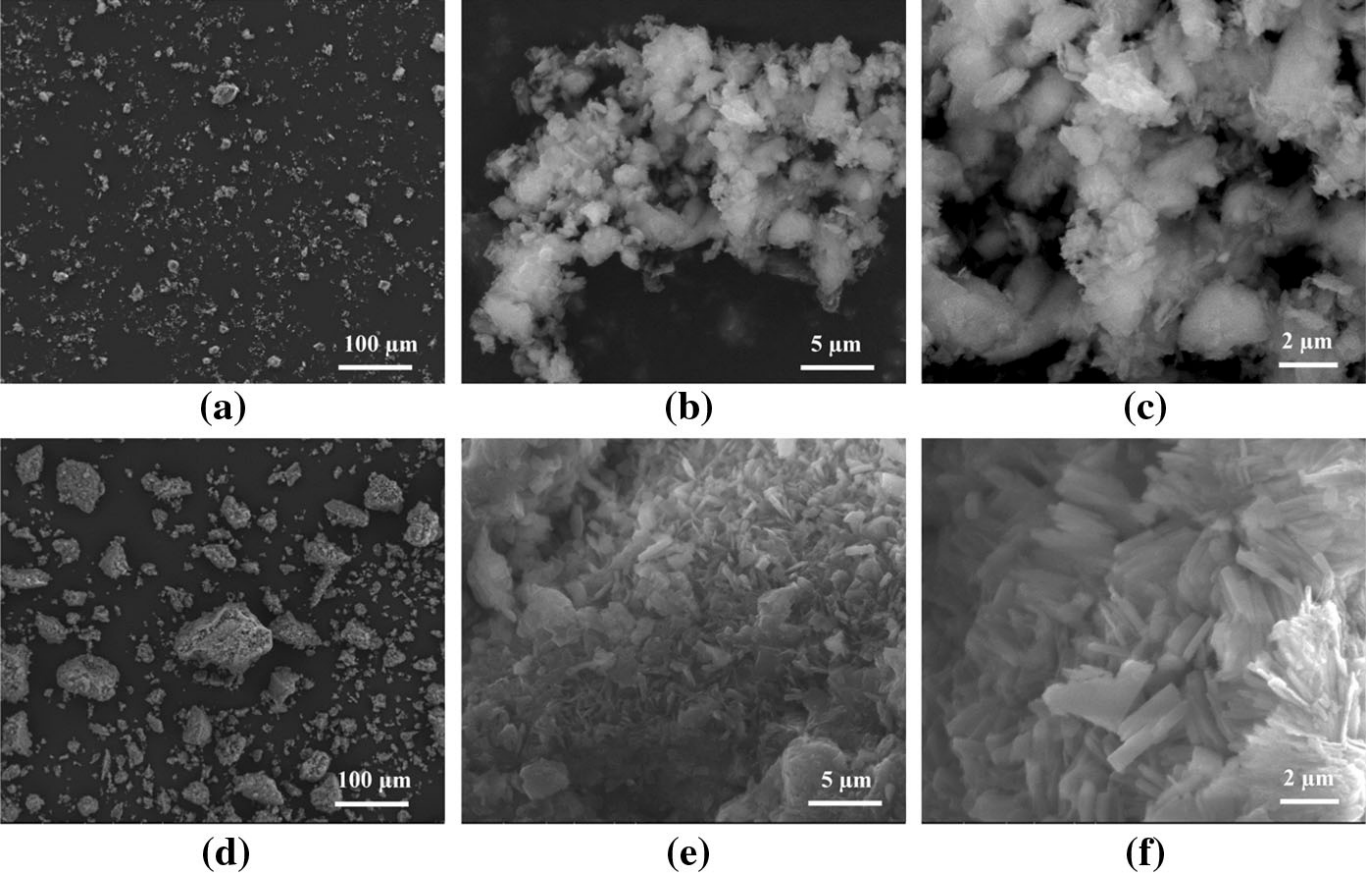
SEM images of the natural Cuban (~ 3 µm) (**a**, **b**, **c**) and Mexican (~ 20–150 µm) (**d**, **e**, **f**) zeolites at different magnifications

The SEM images of the natural Cuban (~ 3 μm) and Mexican (~ 20–150 µm) zeolites at different magnifications are displayed in Fig. 2. Both of these zeolites exhibit contrasting morphologies likely due to their different origin. The Cuban zeolite sample is composed of crystallites without definite morphology (Fig. 2a, b, c). However, the Mexican zeolite sample (Fig. 2d, e, f) shows typical morphologies of clinoptilolite (Cerri et al. 2016). Most of the crystals are well defined in the form of plates and/or laths in the size range of 2–3 μm. It is clearly evident that higher magnifications (Fig. 2c, f) do not show the occurrence of fibrous phases, such as asbestos or erionite, which are known to be carcinogens causing deadly human lung diseases (Carbone et al. 2011). This is a prerequisite for using them as medical device and dietary supplements, respectively, as it was shown previously for Detoxsan^®^ Pulver, which is a mixture of Cuban zeolite (~ 40 µm) with magnesium aspartate (Selvam et al. 2014).

### Particle size analysis

**Fig. 3:**
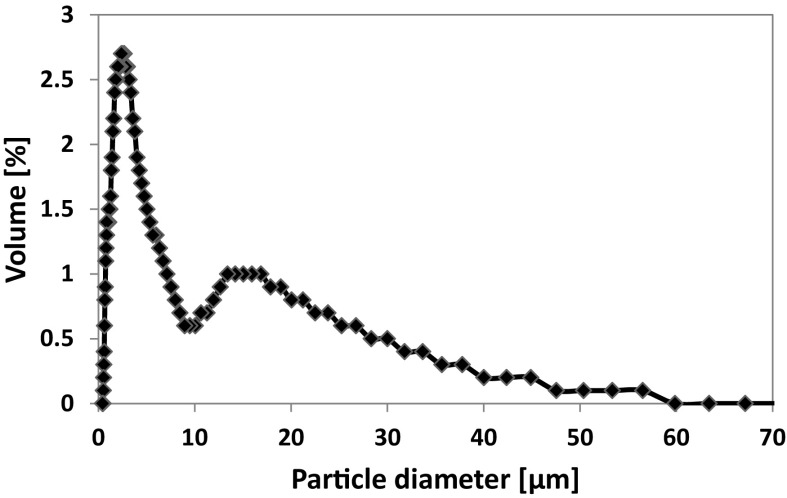
Particle size distribution of the natural Cuban zeolite (~ 3 µm, size class A) by DLS method

The particle size distributions (PSDs) of the fine-grained Cuban (~ 3 μm) zeolite and both the coarse-grained Cuban (~ 40 μm) and Mexican (~ 20–150 μm) zeolites were measured by different methods in order to get reliable results considering their particle sizes (Weiner and Fairhurst 1992). Thus, dynamic light scattering measurement (DLS) was specifically applied in the fine-grained Cuban zeolite (~ 3 μm). The PSD of the Cuban zeolite (~ 3 μm) is shown in Fig. 3. It exhibits a bimodal PSD with a first maximum in the < 3 μm size fraction and a second one in the ~ 16 μm size fraction. According to the data obtained, around 60% of the particles are in the < 3 μm size range. This fine-grained Cuban zeolite (~ 3 μm) is referred hereafter to as size class A.

The PSDs of the coarse-grained Cuban and Mexican zeolites were determined by the dry sieving method according to DIN EN 1015-1: 2007-05 (2007) because of their large particle size. The results are summarized in Table 2 and indicate that PSDs of the coarse-grained Cuban and Mexican zeolites are very different. Moreover, 68.1% of coarse-grained Cuban zeolite is found in < 63 μm fractions. In contrast, more than 75.8% of coarse-grained Mexican zeolite is found in > 63 μm fractions. The specific objective of the present study is to assess and highlight the influences of the particle sizes and textural properties (BET surface areas and pore volumes) of natural zeolites on their histamine-binding capacities. Therefore, for the histamine uptake experiments, we used fractions with similar particle size composition and identified these fractions as size class B and size class C as shown in Table 2.

**Table 2:** Particle size distribution of the Cuban and Mexican zeolites as determined by the dry sieving method

Particle size	Particle size distribution (%) of the Cuban and Mexican zeolites			
(µm)	Cuban (~ 40 µm)	Mexican (as received)	Mexican (< 100 µm)	Mexican (> 150 µm)
Size class	B^a^	–	B^a^	C^a^
< 20	2.2	3.5	9.3	0
20–40	28.9	9.7	25.7	0
40–63	37.0	11.0	29.2	0
63–100	18.6	13.5	35.8	0
100–150	6.9	14.1	0	0
> 150	6.4	48.2	0	100

In accordance with DIN EN 1015-1: 2007-05 (2007)

^a^The size classes B and C of both the zeolites were used for further histamine uptake studies

### N_2_-sorption

The pre-treatment conditions have a pronounced effect on the N_2_-sorption results obtained. It is our experience that inconsistent results were obtained for the samples which were pre-treated under different conditions (105 °C for 24 h, 250 °C for 16 h and 300 °C for 1 h). In particular, natural Cuban zeolite contains about 15% of water as regular constituent (Cervini-Silva et al. 2016). An optimum pre-treatment temperature and sufficient period of time are necessary to make the whole zeolite crystal lattice free of water, prior to the sorption measurements. In the present study, all the samples were pre-treated at 250 °C for 12 h in order to get reproducible results.

The BET surface areas and total pore volumes of Cuban and Mexican zeolites, having different particle sizes, are given in Table 3. Both the Cuban zeolites (size classes A and B) exhibit high BET surface areas (98–119 m^2^ g^−1^) and high total pore volumes (0.199–0.220 cc g^−1^) in comparison to the Mexican zeolites having different particle sizes. This is due to the fact that the mineralogical compositions of both Cuban and Mexican zeolites are very different (see Table 1). As mentioned before (Selvam et al. 2014), the presence of large-pore 12-membered ring zeolite mordenite (pore size: 7.0 × 6.5 Å) along with the medium pore 10-membered ring zeolite clinoptilolite (pore size: 7.5 × 3.1 Å) in Cuban zeolites results in a larger BET surface areas and total micropore volumes. Thus, Mexican zeolites, which contain clinoptilolite (pore size: 7.5 × 3.1 Å) alone, exhibit significantly lower BET surface areas (30–31 m^2^ g^−1^) and total micropore volumes (0.145–0.182 cc g^−1^). This is in agreement with the results (BET surface area: 25 m^2^ g^−1^ and total pore volume: 0.132 cc g^−1^) obtained for the natural zeolite which contains only clinoptilolite (Cerri et al. 2016). Furthermore, tribomechanical micronization does not increase the BET surface area of clinoptilolite (Pavelić et al. 2017). Moreover, concerning both the Cuban and Mexican zeolites, the smaller the size fractions, the higher the total micropore volumes (Table 3). Their significant influences on the histamine-binding capacities will be discussed in the forthcoming section.

**Table 3:** Textural properties and histamine uptake capacities of Cuban and Mexican zeolites having different particle sizes

Zeolite	Particle size class^a^	BET surface area (m^2^ g^−1^)	Total pore volume (cc g^−1^)	Histamine uptake (mg/g zeolite) at different incubation time intervals^b^			
				15 min	30 min	60 min	120 min
Cuban^c^	A	98	0.220	16.3	18.8	19.9	22.4
Cuban^c^	B	119	0.199	15.7	13.7	16.0	15.7
Mexican^d^	B	30	0.182	10.4	9.0	9.5	10.2
Mexican^d^	C	31	0.154	4.1	6.6	5.7	5.7
Mexican^d^	As received	30	0.145	6.0	5.6	6.9	6.5

Histamine uptake studies: 3 g of zeolite, 100 mL double distilled water (pH = 7), 0.3 g histamine, temperature = 36 °C

^a^Classification: Cuban A: ~ 3 µm, Cuban B: ~ 40 µm, Mexican B: < 100 µm, Mexican C: > 150 µm; see Table 2 and Fig. 3

^b^Determined by TG measurements in the temperature range of 350–600 °C

^c^San Andrés

^d^Mina San Francisco

### Histamine uptake

Table 3 summarizes the results of the histamine uptake by natural Cuban and Mexican zeolites having different particle sizes. The histamine uptake rate of the natural zeolites used in the present study seems to be nearly completed within the first 15 min that means the uptake capacities remain nearly stable over a period between 15 and 120 min. Only in the case of the fine-grained Cuban zeolite (size class A) having the largest pore volume, a continuous increase in histamine uptake could be observed. This continuous increase may be due to the better adsorption efficiency of the smaller particles in comparison to the larger ones, indicating that the uptake capacity of histamine depends on the particle size. Large particles seem to have reached more rapidly the maximum histamine uptake capacity than the smaller ones.

Significant differences in histamine uptake could be found between Cuban and Mexican zeolites comparing the nearly identical particle size class B (Table 3). In both samples, the histamine uptake is stable over the period between 15 and 120 min, while the uptake capacity of the Mexican zeolite amounts to only about 64 ± 3% of the Cuban zeolite. This difference seems to be due to the different mineralogical compositions of the two investigated zeolites. The Cuban zeolite contains both the medium pore-sized clinoptilolite and the large pore-sized mordenite, which finally results in a larger pore volume than the Mexican zeolite, which contains only clinoptilolite (Tables 1, 3). Moreover, Mexican zeolite with smaller particles (size class B) also adsorbed significantly more histamine (10.2 mg g^−1^ of zeolite) than the larger ones (size class C; 5.7 mg g^−1^ of zeolite; Table 3).

Rather than the BET surface areas, the pore volumes of the zeolite can be correlated to the enhanced histamine uptake. In size classes B and C, the histamine uptake capacities are nearly stable between 15 and 120 min. Therefore, one can calculate the mean values over this period resulting in 15.3 mg g^−1^ for Cuban (size class B) and 9.8 mg g^−1^ for Mexican (size class B) and 5.5 mg g^−1^ for Mexican (size class C), respectively. Thus, in size class B, the histamine uptake in the Mexican zeolite is about 35% less as well as the pore volume is about 10% less than in the Cuban zeolite. On the other hand, BET surface area of the Cuban one is about 4 times higher than the Mexican zeolite (Table 3). Therefore, the histamine uptake can be better correlated to the pore volume than BET surface area, which allows postulating interdependence. It can be reasoned that pore volume is the most important parameter for high histamine uptake by natural zeolites depending on their mineralogical compositions (pore dimensions of the components) and particle sizes.

Based on these results, it is necessary to keep in mind that the desired goal of zeolite application is for the oral or topical routes. As discussed in our earlier publication (Selvam et al. 2014), it is known that histamine plays an important role in the regulation of production of gastric acid (Schubert 2012). We believe that the histamine-mediated induction of gastric acid may be significantly reduced by natural zeolite through the reduction of histamine level. In this particular case, natural zeolite acts only via its inherent adsorption properties and does not directly influence the regulation processes such as proton pump inhibitors (PPI). Due to various side effects of chronic PPI treatment, there is a tendency to look for alternative therapeutic modalities (Fass 2012). First clinical trials are reported with respect to the reduction of heartburn episodes by potentiated clinoptilolite zeolite as well as the decrease of mucosa erosion under consumption of non-steroidal anti-inflammatory drugs (Potgieter et al. 2014). The authors supposed that the gastro-protective properties of the zeolite seem to be associated amongst others with adsorption properties for biogenic amines like histamine, which plays also an important role as an inducer of food intolerance in sensitive people and may trigger adverse responses (Caballero 2013). Thus, there are many physiological processes regulated by histamine which might be influenced by zeolite and require advanced clinical studies for the necessary zeolite amounts to obtain the desired health benefits.

## Conclusions

Both the zeolites from Cuba and Mexico differ significantly in their mineralogical compositions. In particular, Cuban zeolite contained both clinoptilolite and mordenite as main phases, whereas the Mexican one contained only clinoptilolite. Both of them were pure minerals without any other impurities according to XRD. Due to the large-pore 12-membered ring zeolite mordenite, the BET surface area of the Cuban zeolite (about 100 m^2^ g^−1^) was significantly larger than the Mexican one (about 30 m^2^ g^−1^). For the N_2_-sorption measurement, it is worth to note that it requires a pre-treatment of the zeolite at 250 °C for 12 h in order to eliminate the water present in the crystal lattice as a natural constituent and to get reproducible results. Furthermore, both zeolites were free of visible minerals with a fibrous morphology according to SEM. Both zeolites adsorbed significant amount of histamine, while the Cuban zeolite exhibited a higher histamine-binding capacity than the Mexican one. Comparing the results of both zeolites with their textural parameters clearly indicated that the histamine-binding capacity can be better correlated to the pore volume than BET surface area, which allows postulating interdependence. It can be concluded that pore volume is the most important parameter for high histamine uptake by natural zeolites depending on their mineralogical compositions (pore dimensions of the components) and particle sizes.
